# Associations of CXCL1 gene 5’UTR variations with ovarian cancer

**DOI:** 10.1186/s13048-020-00640-9

**Published:** 2020-04-23

**Authors:** Man Guo, Chao Xu, Yan-Zhe Chen, Qi-Wen Sun, Xin-Ying Zhao, Xin Liu, Yi Yang, Yi-Yan Hu, Fei-Feng Li, Shu-Lin Liu

**Affiliations:** 1 Genomics Research Center, College of Pharmacy (State-Province Key Laboratories of Biomedicine-Pharmaceutics of China), Harbin Medical University, Harbin, China; 2 Department of Gynaecology and Obstetrics of the Second Affiliated Hospital, Harbin Medical University, Harbin, China; 3 Department of Colorectal Surgery of the Second Affiliated Hospital, Harbin Medical University, Harbin, China; 4 Department of Blood Dialysis, Heilongjiang Agricultural Reclamation Bureau General Hospital, Harbin, China; 5 Fifth Hospital Gynecology the City of Xiamen, Xiamen, Fujian China; 6 Translational Medicine Research and Cooperation Center of Northern China, Heilongjiang Academy of Medical Sciences, Hegang, Heilongjiang China; 7 Department of Microbiology, Immunology and Infectious Diseases, University of Calgary, Calgary, Canada

**Keywords:** Ovarian cancer, Chronic inflammation, Chemokines, CXCL1, 5’UTR

## Abstract

**Background:**

There are about 2.4 hundred thousand new cases and 1.5 hundred thousand deaths of ovarian cancer (OC) annually in the world. Chronic inflammation is a risk factor for OC. C-X-C motif chemokine ligand 1 (CXCL1) defects may facilitate inflammation and transactivate EGFR in ovarian cancer, but the precise haplotypes associated with the potential diseases remained largely unknown. In this work, we characterized *CXCL1* gene variations to elucidate their possible associations with OC.

**Methods:**

We analyzed the *CXCL1* gene for 300 OC patients with 400 healthy participants as controls. The statistical analyses and Hardy-Weinberg equilibrium tests of the patients and control populations were conducted using the SPSS software (version 19.0) and Plink (version 1.9).

**Results:**

The variants rs11547681, rs201090116, rs199791199, rs181868085, rs4074 and rs1814092 within or near the *CXCL1* gene were characterized. The genetic heterozygosity of rs11547681 and rs4074 was very high. Statistical analysis showed that the variant rs11547681 in the gene was closely associated with the risk of OC in the Chinese Han population, although this variant was not associated with FIGO stages or pathological grades of the patients.

**Conclusions:**

Rs11547681 in *CXCL1* gene was associated with the risk of OC in the Chinese Han population.

## Introduction

Globally, there are about 2.4 hundred thousand new ovarian cancer (OC) cases and about 1.5 hundred thousand deaths caused by this disease annually [1]. OC is the 7th most common and 5th most lethal cancer among women worldwide [2]. The incidence rates of OC are highest in northern and eastern Europe, and lowest in Asia and Africa [1]. In the past decades, however, the morbidity has considerably decreased in many previously high incidence countries but, in contrast, is increasing in some low incidence countries, such as China [1, 3].

The 5-years survival rates of OC range from 30.3 to 44.1%, depending on the specific subtypes and stages at the time of diagnosis or treatment [4, 5]. Early detection and treatment of this disease can significantly increase the survival rate of OC, but, due to the lack of specific signs or symptoms at early stages, this disease is usually diagnosed only at late stages [6]. The recurrence rate of this disease is also very high, often leading to death of the patient, who might have achieved a clinical complete remission after primary therapy [5, 6].

So far, several genetic and non-genetic factors have been found to be associated with OC. Women with affected first-degree relatives usually have higher risks for OC [7], and women, who have relatives diagnosed with OC below 50 years old, have even higher OC risks [8]. Many gene mutations have been associated with sporadic OC patients, including BRCA1 and 2 [9], BRIP1 [10] and RAD51 [11]. Oral contraceptive use and tubal ligation seem to be protective factors [12, 13], whereas older menopausal ages, obesity, menopausal hormone therapy use, a history of endometriosis and smoking are all risk factors [14–16].

Chronic inflammation, such as those caused by asbestos or talc exposure, endometriosis or pelvic inflammatory diseases, has also been suggested to be a risk factor for OC [17, 18]. On the other side, anti-inflammatory medications, e.g., acetaminophen and low-dose of aspirin are protective factors for OC [19, 20]. Immunotherapies, including immune checkpoint blockade and cancer vaccines, also have many special roles in immune recognition and immune regulation of the OC cells [21].

Chemokines expressed in tumor or stromal cells may facilitate tumor angiogenesis and in the meantime suppress immune-mediated tumor elimination, and as a result are associated with cancers [22–24]. C-X-C motif chemokine ligand 1 (CXCL1) is a member of the CXC subfamily of chemokines and an oncogenic factor in many cancers [25, 26]. It can transactivate EGFR in OC through binding to the G-protein coupled receptor CXC receptor 2 (CXCR2) [27]. Abnormal expression of CXCL1 is associated with many tumors [28], but the associated haplotypes remained largely unknown.

In this work, we investigated variants in the *CXCL1* gene for their associations with the risk of OC in the Chinese Han population. We found that variant rs11547681 was associated with ovarian cancer and demonstrated the 5’UTR for the functions of CXCL1.

## Materials and methods

### Study population

A total of 300 sporadic OC cases and 400 normal controls (Table 1) were assembled for this study at the Department of Gynaecology and Obstetrics and Medical Examination Center of the Second Affiliated Hospital of Harbin Medical University, Harbin, China. From each participant, we obtained a written informed consent. This work has been reviewed and approved by the Ethics Committee of Harbin Medical University. We also confirmed that all experiments were performed in accordance with relevant guidelines and regulations and were consistent with the 1975 Declaration of Helsinki.

**Table 1:** Clinical characteristics of study population

***Parameter***	***CRC***	***Control***	***F***	***t***	***P***	***95%CI***	
						***Up***	***Low***
***Sample (n)***	300	400	–	–	–	–	–
**Age (years)**	50.39 ± 12.21	49.68 ± 7.66	9.977	0.639	0.523	−1.47926	2.90141

Data are shown as mean ± SD; between the two groups, there were no statistical differences of the age and gender composition

Medical histories of the enrolled participants were recorded in detail, and all the participants received physical examinations. The diagnostic criteria for sporadic OC patients were those of FIGO (Federation International of Gynecology and Obstetrics) and the patients had no history of other systemic abnormalities or previous tumor or familial history of tumor. The exclusion criteria for the control participants were any diseases or systemic abnormalities.

### DNA analysis

We used standard protocols to extract the genomic DNA from the peripheral blood leukocytes as described previously [29]. The *CXCL1* gene was amplified by PCR with the primers shown in Table 2. PCR products were sequenced using standard protocols [30, 31] for genotype analysis.

**Table 2:** PCR primers used for *CXCL1* gene sequence analysis

***Exon***	***Forward primer***	***Reverse primer***	***Size (bp)***	***Tm (°C)***
***Exon1***	GCGGGCTGCATCAGTGGA	CGGGACTTACATGACTTCGGT	595	59.8
***Exon2***	CTGCTGCTCCTGCTCCTGGTA	GGAAGGGAATCTCGTGAGGC	370	59.4
***Exon3***	AAACCGAAGTCATGTAAGTCC	CAATAATCCCAATTTCTAGTCC	336	54.0
***Exon4a***	TTAGAGGTCCCTGCCACA	ATTCCCCTGCCTTCACAA	629	52.2
***Exon4b***	TGCAACATGCCAGCCACT	ATAGCAAATTGAACACCC	460	50.0

### SNP genotyping and statistical analysis

The variations within or near the *CXCL1* gene were determined for the 300 sporadic OC cases and 400 normal controls. The DNA regions were amplified and the PCR products were sequenced to determine the genotypes; two researchers conducted the measurements independently. Overall OC genetics correlation analysis was also conducted. As previously reported, the statistical analyses and Hardy–Weinberg equilibrium tests of the patients and control populations were conducted [29, 32–34].

## Results

### Clinical data

The clinical diagnosis of all the participants was confirmed by specialists in Department of Gynaecology and Obstetrics in the Second Affiliated Hospital of Harbin Medical University, Harbin, China. These OC patients had no history of other systemic abnormalities or previous tumor or familial history of tumor. All the OC patients (*n* = 300, female, average age was 50.39 years, the min and max age were 18 and 81 respectively) and normal controls (*n* = 400, female, the average age was 49.68, the min and max age were 31 and 67 respectively) were recruited specifically for this study. There were no statistical differences in age composition between the two groups (Table 1).

### SNP gene analyses

**Fig. 1:**
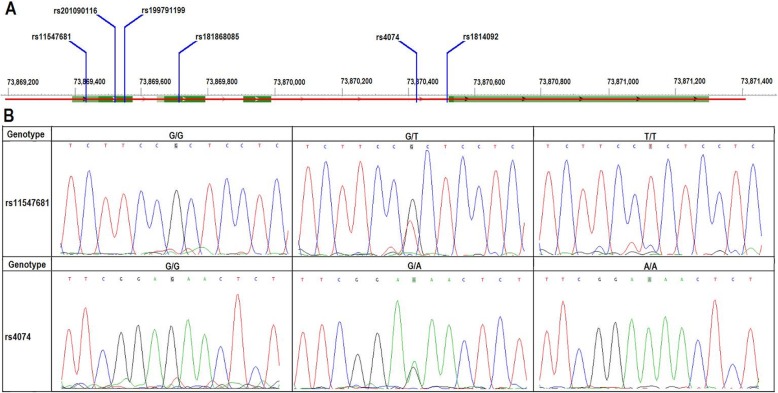
Schematic diagrams and DNA sequence chromatogram of SNPs in *CXCL1* gene. **a**: locations of rs11547681, rs201090116, rs199791199, rs181868085, rs4074 and rs1814092 within the *CXCL1* gene; **b**: DNA sequence chromatogram of the three polymorphisms identified in the *CXCL1* gene in all the population used for disease-association analyses

In order to test the hypothesis that germline common genetic variants in *CXCL1* gene may be associated with the susceptibility to OC, we extracted the genomic DNA from the peripheral blood leukocytes of the participants and sequenced the *CXCL1* gene to detect SNPs. We found six SNPs distributed on the gene, including rs11547681, rs201090116, rs199791199, rs181868085, rs4074 and rs1814092 (Fig. 1a). Analysis of these SNPs showed that the genetic heterozygosity of rs11547681 and rs4074 was very high (Fig. 1b), whereas that of rs201090116, rs199791199, rs181868085 and rs1814092 was very low and were excluded from further analysis.

### Polymorphism-disease association analyses

To test the hypothesized associations between *CXCL1* variations and OC, we conducted analyses on the SNPs and found the variant rs11547681 within the 5’UTR of the gene was associated with the risk of OC in the Chinese Han population (Tables 3 and 4). Further, we analyzed the Hardy–Weinberg equilibrium test for the study population groups, and it was in line with equilibrium (Table 5).

**Table 3:** The genotype and allele frequency of variations in 300 Chinese Han ovarian cancer patients and 400 normal controls

***SNP***	***Group***		***Genotype frequency (%)***			***Allele frequency (%)***	
*rs11547681*	Genotype		G/G	G/T	T/T	G	T
	OC	300	185 (61.7)	107 (35.7)	8 (2.7)	477 (79.5)	123 (20.5)
	Controls	400	294 (73.5)	94 (23.5)	12 (3.0)	682 (85.3)	118 (14.8)
*rs4074*	Genotype		G/G	G/A	A/A	G	A
	OC	300	84 (28.0)	153 (51.0)	63 (21.0)	321 (53.5)	279 (46.5)
	Controls	400	120 (30.0)	207 (51.8)	73 (18.3)	447 (55.9)	353 (44.1)

Note: *OC* Ovarian Cancer

**Table 4:** rs11547681 variations within 5’UTR of the *CXCL1* gene associated with risk of ovarian cancer in Chinese populations

***Variations***	***Type***	***Pearson Chi-square***				***Risk***		
		Value	Min count	df	Asymp. Sig. (2-sided)	Value	95% CI-low	95% CI-up
*rs11547681*	Genotype	12.412	8.57	2	0.002^a^	–	–	–
	Allele	7.954	103.29	1	0.005^a^	0.671	0.508	0.886
*rs4074*	Genotype	0.921	58.29	2	0.631	–	–	–
	Allele	0.781	270.86	1	0.377	0.909	0.735	1.124

^a^: statistically significant

**Table 5:** Hardy-Weinberg equilibrium test for the study population groups

***SNPs***	***Genotype***		***H-W equilibrium Testing***		
	Homo/Hetero/Homozygote		O (HET)	E (HET)	P
*rs11547681*	OC	8/107/185	0.3567	0.3259	0.1541
	Controls	12/94/294	0.2350	0.2515	0.2292
*rs4074*	OC	63/153/84	0.5100	0.4975	0.7281
	Controls	73/207/120	05175	0.4931	0.3618

Note: *OC* Ovarian Cancer

The genotype frequencies in the two groups were also analyzed by three genetic models (trend, dominant and recessive), and we found that the rs11547681 was associated with the risk of OC in trend and dominant models (Table 6). For the variant rs4074, we did not find statistical significance between the OC and control groups (chi-square tests; trend, dominant and recessive models). We compared the genotype frequency of the rs11547681 and rs4074 in the two groups and the data from the HapMap HCB population, but did not find the genotype frequency data of rs11547681 in the HapMap HCB population.

**Table 6:** SNP rs11547681 within *CXCL1* gene associated with the risk of ovarian cancer

***SNPs***	***Value***	***Trend model***	***Dominant model***	***Recessive model***
*rs11547681*	ChisQ	8.0140	11.1100	0.0686
	P	0.0046^a^	0.0009^a^	0.7933
*rs4074*	ChisQ	0.8121	0.3321	0.8282
	P	0.3675	0.5644	0.3628

^a^: statistically significant

### Clinical features comparative analysis

We also compared the clinical characteristics between the wild type, heterozygous variant and homozygous variant groups of the OC patients, but did not find statistically significant differences between the three groups in FIGO stages and pathological grades (Table 7).

**Table 7:** Comparative analysis of clinical features between wild type, heterozygous variation and homozygous variation groups

Clinical Index	Wild Type	heterozygous variation	homozygous variation	Chi-Square Test
**TNM Stage (I/II/III/IV)**	59/32/92/2	30/23/54/1	2/1/5/0	*P* = 0.956
**Pathological Grades(H/M/L/Non)**	101/15/36/33	54/7/31/15	5/0/3/0	*P* = 0.375

*H* Pathological high Grade; *M* Pathological moderately Grade; *L* Pathological low Grade; *Non* No pathological grade

## Discussion

In this study, we found that the SNP rs11547681 within the 5’UTR of *CXCL1* gene was associated with OC. The microenvironment of tumors is an important factor in modulating cancer development, especially in organs that communicates with the outside environment [35, 36]. The cells surrounding those tumors usually release some factors, such as growth or inflammatory factors, which may regulate inflammation or progression of tumors [37, 38].

The human chemokines have strong activities on tumor cells, especially in cross talk of tumor cells and their host microenvironment [39]. CXCL1 is one member of the chemokines, which is a proinflammatory mediator in many inflammatory diseases. CXCL1 promotes and exacerbates growth and progression of many tumors [40]. By activating CXCR2, CXCL1 is associated with cancer cell growth and proliferation, tumor angiogenesis and metastasis [41, 42]. In tumor therapies, CXCL1 is also responsible for resistance of the cancer cells to several chemotherapeutic drugs [43].

In the OC cells, over-expression of the CXCL1 factor promotes the abilities of cellular proliferation and invasion in vitro [27, 44]. Progesterone and calcitriol can inhibit ovarian and endometrial cancer cell growth by attenuating the functions of CXCL1; if the expression of CXCL1 is reduced, the inhibitory effect of the two agents is also abrogated [45]. Conversely, when the expression of CXCL1 is increased, the activation of metastasis promoting gene p65 is also increased in OC cells [45]. The serum CXCL1 may be a novel tumor marker for OC diagnosis [46]. In this study, we found that the SNP rs11547681 in the *CXCL1* gene was associated with OC. This finding provides novel insights into the special roles of CXCL1 factor for the pathogenesis, diagnosis and therapies of ovarian cancer.

Many chemokines exist as monomers or dimers in vivo. They function by binding to tissue glycosaminoglycans (GAGs) heparan sulfate (HS), chondroitin sulfate (CS) or dermatan sulfate (DS) [47, 48]. GAGs bind to a diversity of protein classes [49], so in order to interact with GAGs, the sequence of chemokine must determine the selectivity, affinity and geometry [50]. CXCL1 belong to the CXC chemokines subset, characterized by the N-terminal‘ELR’motifs [51]. The amino acid residues located within the N terminal loop and C terminal helix of CXCL1 factor mediate HS binding, and the participation of other residues may result in a very different binding geometry for CXCL1 [50]. The SNP rs11547681 associated with OC is located within the 5’UTR of CX*CL1* gene. The 5’UTR and 3’UTR sequences regulate expression of genes [52, 53]. The 5’UTR sequences of gene bind with miRNAs and may be involved in gene expression, protein translation or disease pathogenesis [54]. In previous studies, we reported SNPs within the 5’UTR or 3’UTR sequences that are associated with diseases [31, 32, 55]. The results of this work further emphasized the important roles of 5’UTR sequences for CXCL1 factor functions.

In conclusion, we demonstrated the associations of *CXCL1* variants rs11547681 with the risk of ovarian cancer in the Chinese Han population and updated our understanding on 5’UTR for CXCL1 functions, providing new information on the pathogenesis of cancers especially ovarian cancer.

## Data Availability

The datasets used in the present study are available from the corresponding authors with reasonable requests.
